# Five-year outcomes of digital diabetic eye screening in individuals aged 80 and 85 years

**DOI:** 10.1038/s41433-023-02577-x

**Published:** 2023-05-20

**Authors:** Kevin Thomas, Nichola Albutt, Aisha Hamid, Helen Wharton, Sarita Jacob

**Affiliations:** 1 College of Medical and Dental Sciences, University of Birmingham, Birmingham, UK; 2 Birmingham, Solihull and Black Country Diabetic Eye Screening Programme, Birmingham, UK; 3 University Hospitals Birmingham, Birmingham, UK; 4 College of Health and Life Sciences, Aston University, Birmingham, UK

**Keywords:** Public health, Health care economics

## Abstract

**Objective:**

To assess the incidence of referable diabetic retinopathy (DR) in patients aged 80 and 85 years to determine whether screening interval can be extended safely in this age group.

**Methods:**

Patients who were aged 80 and 85 years when they attended digital screening during April 2014–March 2015 were included. Screening results at baseline and over the next four years were analysed.

**Results:**

1880 patients aged 80 and 1105 patients aged 85 were included. Patients referred to hospital eye service (HES) for DR ranged from 0.7% to 1.4% in the 80-year-old cohort over 5 years. In this cohort a total of 76 (4%) were referred to HES for DR, of which 11 (0.6%) received treatment. Over the course of the follow up (FU), 403 (21%) died. In the 85-year-old cohort, referral to HES for DR each year ranged from 0.1% to 1.3%. In this cohort a total of 27 (2.4%) were referred to HES for DR, of which 4 (0.4%) received treatment. Over the course of follow-up 541(49%) died. All treated cases were for maculopathy in both cohorts and there were no cases of proliferative diabetic retinopathy requiring treatment.

**Conclusion:**

This study showed that the risk of progression of retinopathy is quite low in this age group and only a small proportion of patients developed referable retinopathy requiring treatment. This suggests relooking at the need for screening and ideal screening intervals in patients aged 80 years and over with no referable DR as they can be potentially classed as a group with low risk of sight loss.

## Introduction

Diabetic retinopathy (DR) is a common cause of blindness in the working age adult population worldwide [1]. Individuals who have diabetes have an increased risk of developing retinopathy. In 2012, the population of the UK with diabetes was approximately 3.8 million [2]. Diabetes UK has reported that more than 4.9 million people in the UK have diabetes in 2020 with a predicted rise to 5.5 million by 2030. Epidemiological data projections show that the prevalence of diabetes will prove to be a significant public heath burden, especially amongst the elderly population [3].

In 2003, the NHS Diabetic Eye Screening Programme was established in England. 15 years later, 82.7% of diabetics in the UK were screened through the programme [4]. The screening programme uses two 45-degree field mydriatic digital photography. This results in images that can be evaluated for diabetic grading by trained retinal graders [5]. A 2014 review by Liew, which compared blindness certifications in the UK in 1999–2000 and 2009–2010, showed that for the first time in 50 years, diabetic retinopathy was not the most prominent cause of blindness amongst working age adults in England [6]. Following this, in 2018/19 there has been a reduction of 2% (of all certifications) in the number of individuals that have diabetic eye disease as their main cause of blindness. This reduction has been centred around age groups of 35 and older, with those below this age showing minimal change [7].

Research suggests that DR is not associated with advancing age, but has a strong association with the type and duration of diabetes [8, 9]. The prevalence and severity of diabetic retinopathy in the elderly aged 70 years and over has been found to be relatively low [10, 11]. Moreover, it has been noted that progression from background to proliferative retinopathy is less likely in the elderly population [12].

The NHS Diabetic Eye Screening programme aims to annually screen anyone with diabetes aged 12 years or older. Other screening programmes within the UK have an upper age limit, but this has not been considered for diabetic eye screening yet [13]. In September 2020, Public health England (PHE) has introduced extended screening intervals for those with no signs of retinopathy who will now be screened in 24 months instead of annually. This new guidance is targeted at individuals with the lowest risk of diabetes related sight loss. The main aim of our study is to assess the progression of diabetic retinopathy over 5 years in patients aged 80 and 85 years to see if they can be classed as a low-risk category for sight loss which in turn would make them suitable for appropriately extended screening intervals.

## Materials & methods

Patients were selected from the Digital Healthcare database of Birmingham, Solihull and Black Country Diabetic Eye Screening Programme with audit approval from CARMS (Clinical audits and registries management service) at UHB (University Hospitals Birmingham) complying with the Declaration of Helsinki. All patients who were aged 80 and 85 years when they attended screening during April 2014–March 2015 were included in this retrospective audit. Screening results at baseline and over the next four years were analysed along with the patient demographics. Diabetic retinopathy results, other ocular findings, attendance rates and referral pattern to eye clinics were documented. Within the study, referrable DR was classed as maculopathy, pre-proliferative or proliferative changes. All referable maculopathy grades (M1) were included in the referable DR but only true positive cases of maculopathy were referred to eye clinics and others were monitored in Digital surveillance (DS) clinics and appropriate outcomes selected, based on findings at subsequent visits. It should be noted that criteria for referable maculopathy also included patients with vision of 6/12 or less with any microaneurysm or haemorrhage within 1DD of the fovea. The clinical outcomes for patients referred to eye clinics with referable DR were obtained from the hospital records and treatment with laser or injections noted.

## Results

From the period of April 2014–March 2015, the database showed 115,606 patients who were screened. 1880 patients aged 80 years and 1105 patients aged 85 years making a total of 2985 patients were included who met the criteria of the study being 80 and 85 years respectively at the time of the study. The average age of the first ever screen for both cohorts was 73 years. The demographics of the patients for the 80 cohort were 60% Caucasian, 21% Asian and 11% Black and remaining not known. 961 (51%) were male and 919 (49%) were female in this cohort. The demographics of the patients for the 85 cohort were 55% Caucasian, 7 % Asian, 5% Black and remaining not known. 531(48%) were male and 574 (52%) were females in this cohort.

**Fig. 1:**
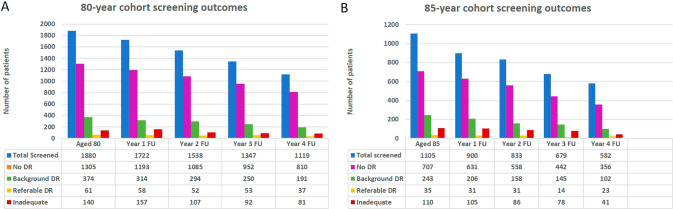
5 year outcomes for Digital screening. Bar charts showing data for 80 -year cohort (**A**) and 85- year cohort (**B**).

Figure 1 shows detailed screening outcomes for both cohorts. The low rates of referable DR can be noted in both groups and unassessable images (inadequates) on standard photography were referred to slit lamp clinics. The unassessables due to dense cataract on slit lamp were referred to cataract clinics in HES and no referable DR was picked up in this group from the available follow up data in HES.

Table 1 shows the data for rates of referable retinopathy, referral rates to eye clinics and treatment rates at baseline screening and subsequent years. The 80-year cohort showed referable DR rates between 3.2 and 3.9% but actual referral to HES ranged between 0.7 and 1.4% with a low treatment rate of 0.6%. The same pattern was reflected in the 85-year cohort where referable DR rates ranged between 2 and 3.9%. The actual referral to HES was noted to be 0.1% to 1.3% here with an even lower treatment rate of 0.4%. Patients with referable DR not referred to HES were the ones with suspect maculopathy who were seen in the Digital surveillance (DS) clinics. These patients were monitored in DS clinics in 6 months if stable, back to annual recall if improved and referred to HES if worsened. Having OCT in our DS clinics helped to identify true maculopathy and diabetic macular oedema for referral to HES. False positives based on vision were sent back to annual recall and minimal dry maculopathy monitored in DS until they resolved or referred to HES if worsened.

**Table 1:** Yearly referral and treatment rates for DR.

	Age—80 years				Age—85 years			
	Total	Referable DR (%)	Referred to HES (%)	Treated (%)	Total	Referable DR (%)	Referred to HES (%)	Treated (%)
Baseline screen	1880	61 (3.2%)	26 (1.4%)	2 (0.1%)	1105	35 (3.2%)	14 (1.3%)	3 (0.3%)
1-year FU	1722	58 (3.4%)	12 (0.7%)	1 (0.1%)	900	31 (3.4%)	2 (0.2%)	0
2-year FU	1538	52 (3.4%)	11 (0.7%)	3 (0.2%)	833	31 (3.7%)	6 (0.5%)	0
3-year FU	1347	53 (3.9%)	18 (1.3%)	4 (0.3%)	679	14 (2.0%)	1 (0.1%)	0
4-year FU	1119	37 (3.3%)	9 (0.8%)	1 (0.1%)	582	23 (3.9%)	4 (0.4%)	1 (0.2%)
Total over 5 years	1880	NA	76 (4.0%)	11 (0.6%)	1105	NA	27 (2.4%)	4 (0.4%)

**Fig. 2:**
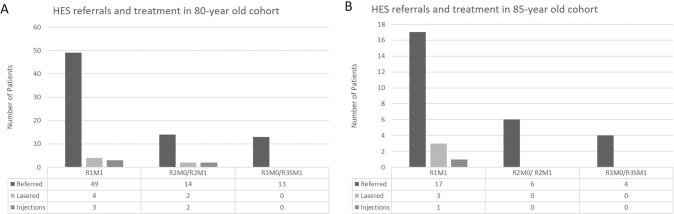
Pattern of referrals to eye clinics and treatment received. (**A**) showing data for the 80-year cohort and (**B**) depicting the 85-year cohort.

As depicted by Fig. 2, in the 80-year cohort, a total of 76 (4%) patients were referred to HES over the five years and 11 (0.6) patients received treatment with macular focal laser and injections for maculopathy. R2 referrals were all stable or improved and none progressed to R3. The patients who were referred for R3 were all found to be either previously treated stable R3 or false positives who were downgraded to R2/ R1 in HES. None of these patients required any treatment and were monitored in HES or discharged back to the screening programme. In the 85-year cohort, a total of 27 (2.4%) patients were referred to HES over the five years and 4 (0.4%) patients received treatment with macular focal laser and injections for maculopathy. All R2 referrals were deemed stable or improved to R1 when seen in HES. Of the four R3 referrals, three were stable treated ones and one was a false positive who was downgraded and all were discharged back to the screening programme. It was only maculopathy that required treatment in both these cohorts and nobody in this age group worsened to proliferative retinopathy over the follow up period and none required pan retinal photocoagulation.

**Fig. 3:**
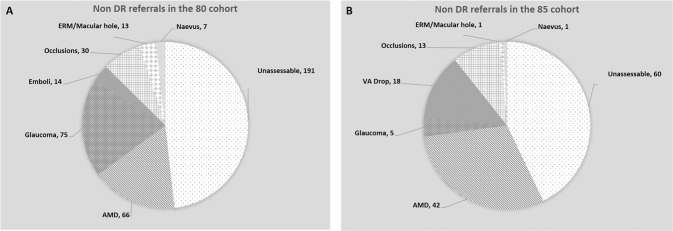
Reasons for non-DR referrals to Hospital Eye Service (HES). (**A**) Showing data for the 80-year cohort and (**B**) depicting the 85-year cohort.

Figure 3 shows the non-DR referrals to HES over the 5 years in both these cohorts. In the 80-year cohort, 396 (21%) patients were referred to HES during the follow up period for other eye conditions and similarly in the 85-year cohort, a total of 140 (13%) patients were referred for other eye conditions. The detailed break up for this can be noted in the Fig. 3 which shows the various non-DR eye conditions for which these patients were referred to HES over the follow up period. Unassessable images due to cataract accounted for the highest proportion of referrals in both the cohorts.

It was also noted that, 403 (21%) patients in the 80-year cohort and 541 (49%) patients in the 85-year cohort died over the course of follow up. This was reflected in the progressive reduction in the total numbers screened in each subsequent year of follow up for both cohorts and can be noted in Fig. 1.

## Discussion

This study showed that the risk of progression of retinopathy over 5 years is quite low in this elderly age group (80–90 years) and only a small proportion of patients developed referable retinopathy requiring any treatment. This raises the question on the need for annual diabetic eye screening in this age group. Tye et al. have evaluated results of digital DR screening in those aged 90 or over and found that only 1% of the patients were referred to eye clinic for DR, 0.5% required treatment with laser and majority of referrals to eye clinic were non-DR related [13]. Our study has also shown comparable data where annual referral rates to HES for DR and treatment rates in both the cohorts remain very low over their five-year follow up. The study conducted by Hirvelä et al. showed that despite a high prevalence of diabetes mellitus in the elderly, the proportion of vision threatening DR and risk of developing proliferative diabetic retinopathy was low in people aged 70 years and over [14]. Other studies have also shown that proliferative retinopathy was practically not detected in people over 80 years [12]. Our study also confirms that there was no progression to proliferative retinopathy in both the cohorts screened over 5 years. The referrals for proliferative retinopathy noted in both groups in our study were all previously treated stable ones or false positive referrals who were subsequently downgraded in the eye clinics. Pan retinal photocoagulation was not needed for any of the patients referred to HES as noted from their electronic patient records over 5 years.

In line with other published data [13, 14] we have also identified that annual DR screening is very helpful in detecting a wide variety of non-DR related routine and sight threatening conditions. 13–21% of patients in this age group were found to have other eye conditions that needed assessment in eye clinics. Unassessable images referred to HES were predominantly due to cataract as noted in other studies. This clearly suggests that it would be beneficial for patients in this age group to visit their optician regularly to enable identification of non-DR related eye diseases and get promptly referred to appropriate eye clinics without any delay.

Cheyne et al. have recently concluded that the incidence of sight threatening diabetic retinopathy confirmed by slit lamp biomicroscopy was less than 2% in a 11-year cohort study from a large urban diabetic screening programme in people aged 12 years and over [15]. This supports consideration of extended screening intervals for people at low risk which is what we are proposing for people aged 80 years and over.

This study has looked at two specific age groups, 80 and 85 year olds and their 5 year follow ups from baseline, the purpose of which was to get the referable DR and treatment rates every year between the age of 80 and 90. The limitation here is the inability to include the entire cohort of patients between the age of 80 and 90 which would have validated the study even more but we had a large number of patients in both cohorts chosen and hence can be considered a good representation of this age group. It was noted that the HES referral and treatment rate was very low, but a small proportion of patients required treatment which forms the basis for our suggestion to increase the DR screening interval to 5 years for persons aged 80 years and over with no referable DR to start with. We are aware that this group could be self-selected as survivors with relatively good health and fewer systemic comorbidities and complications of diabetes. But we have demonstrated that this patient cohort over the age of 80 showed low risk of progression of retinopathy over 5 years and lower risk of sight threatening DR. Further similar studies once the screening interval has been extended to five years with an even larger sample size including the entire population aged 80 years and over in a large screening programme like ours would be helpful to further validate our study with regards to progression of retinopathy. It may then be possible to look at stopping systematic screening in this age group if further studies can confirm this observation. Patients can then be encouraged to visit their opticians regularly to help pick up any sight threatening disease especially non-DR related which seems to be the main reason for referral to HES in this age group. Accepting that relying on patients to present to optometry services may lead to some delay in diagnosis, we propose that patient letters should automatically go out from the screening programme annually to those individuals and their GP’s (General Practitioners) where screening has been extended. The letter will clearly advice the individual and GP that they have a small risk of developing sight threatening retinopathy and should be visiting their optician annually for a check until they get the next invite for diabetic eye screening in 5 years’ time. Formal documented correspondence from the screening programme every year will serve as a good stimulus for patients to attend the optometry services. GP’s being copied in can also serve as the connecting link to ensure that their patients see the optician within 2 years of last diabetic eye screening. Empowering GPs would be a great way to ensure the feasibility and safety of this plan to ensure that the few cases that may develop referable DR do not get missed. This clearly requires careful planning and systematic evaluation to ensure long term safety. The digital data base did not have data for the type and duration of diabetes or HbA1C levels for all patients which would have helped further in deciding on the risk of developing sight threatening disease.

We also noted that a significant proportion of patients died during the course of follow up reflecting on the number screened each year which was lower due to death along with other medical conditions and cognitive impairment which prevented screening attendance.

The 80 and 85-year-old cohort alone in a single year’s screening data from a single large urban screening programme as in this study (*n* = 2985) contributed to 2.6% of the screening workload (*n* = 115,606). This figure may be significantly higher if we look at the entire cohort of diabetic patients over the age of 80 years nationally.

In summary our data suggests that referable DR to HES and treatment rates for DR are low in people aged 80 years and over. Progression to proliferative retinopathy was not noted in this age group and previously treated proliferative retinopathy and maculopathy has remained stable. Treatment for maculopathy was needed only in a small proportion of patients and this can still be picked up with extended screening intervals. Screening attendance rates were found to decrease significantly with advancing age due to reasons such as death and other diseases making them no longer suitable for screening. This study supports and calls for a review of current guidance. Screening intervals need to be relooked at in this 80 plus cohort with no referable retinopathy. Extension of screening interval to five years with optional or mandatory screening with optician as needed in between is a suggestion which would help to pick up any other sight threatening disease and maculopathy as well if any during the extended interval. This would be relevant and cost effective in view of the current national recommendation to increase the screening interval to two years in people with low risk of sight loss. Further studies looking at the entire cohort of diabetics over the age of 80 years with systematic evaluation and careful planning by the UK national screening committee would be needed before any recommendations can be considered.

## Summary

### What was known before

- Previous studies with smaller numbers have looked at diabetic screening in persons over 70 years and those over 90 years more recently to assess the value of screening in these age groups.
- All the published studies so far have looked at data and outcomes for these patients at the time of screening episode, but none have looked at five-year follow up and outcomes for a large cohort of patients between the age of 80 to 90 years to validate the observation of low rates of referable DR and treatment needed.

### What this study adds

- This study has shown that referable DR, referral rates to eye clinics and subsequent need for treatment for DR remains very low in this age group of 80–90 years which has been validated by a five year follow up of the same large cohort of patients.
- This study also confirms that the risk of sight threatening diabetic retinopathy is very low in this age group, but further studies are warranted to suggest stopping screening or introducing an upper age limit for screening in this group at present.
- We suggest increasing the screening intervals for persons aged 80 years and over with no referable DR to 5 years to start with as this is a low-risk group. This is a relevant and cost-effective suggestion without compromising patient safety, considering the current national recommendation for low-risk patients and recent increase in screening interval to two years in people with low risk of sight loss.

## Data Availability

The data that support the findings of this study are available from the authors but restrictions apply to the availability of this data, which was used with permission from the Birmingham, Solihull and Black Country Diabetic Eye Screening Programme (BSBC DESP) for the current study, and so it is not publicly available. Data is available from the authors upon reasonable request and with permission from the information governance and data protection team of BSBC DESP.
